# Atmospheric benzenoid emissions from plants rival those from fossil fuels

**DOI:** 10.1038/srep12064

**Published:** 2015-07-13

**Authors:** P.K. Misztal, C.N. Hewitt, J. Wildt, J.D. Blande, A.S.D. Eller, S. Fares, D.R. Gentner, J.B. Gilman, M. Graus, J. Greenberg, A.B. Guenther, A. Hansel, P. Harley, M. Huang, K. Jardine, T. Karl, L. Kaser, F.N. Keutsch, A. Kiendler-Scharr, E. Kleist, B.M. Lerner, T. Li, J. Mak, A.C. Nölscher, R. Schnitzhofer, V. Sinha, B. Thornton, C. Warneke, F. Wegener, C. Werner, J. Williams, D.R. Worton, N. Yassaa, A.H. Goldstein

**Affiliations:** 1 University of California Berkeley, Environmental Science, Policy, and Management, Berkeley, CA 94720, USA; 2 National Center for Atmospheric Research, Atmospheric Chemistry Division, Boulder, CO 80301, USA; 3 Lancaster Environment Centre, Lancaster University, Lancaster LA1 4YQ, UK; 4 Institut IBG-2, Phytosphäre, Forschungszentrum Jülich, 52425 Jülich, Germany; 5 Department of Environmental Science, University of Eastern Finland, 70211 Kuopio, Finland; 6 CIRES, University of Colorado, Boulder CO 80309 USA; 7 University of Colorado, Department of Ecology and Evolutionary Biology, Boulder, Colorado 80309 USA; 8 Council for Agricultural Research and Economics, Research Centre for the Soil-Plant System, Rome, Italy; 9 University of California Berkeley, Department of Civil and Environmental Engineering, Berkeley, CA 94720, USA; 10 Yale University, Chemical and Environmental Engineering, New Haven, CT 06520, USA; 11 ESRL-NOAA, Chemical Sciences Division, Boulder CO 80305 USA; 12 Pacific Northwest National Laboratory, Atmospheric Sciences and Global Change Division, Richland, WA, USA; 13 Washington State University, Department of Civil and Environmental Engineering, Pullman, WA, USA; 14 University of Innsbruck, Institute for Ion Physics and Applied Physics, 6020 Innsbruck, Austria; 15 Estonian University of Life Sciences, Department of Plant Physiology, Tartu, Estonia; 16 Lawrence Berkeley National Laboratory, Climate Sciences Department, Berkeley, CA 94720, USA; 17 University of Innsbruck, Institute of Atmospheric And Cryospheric Sciences, 6020 Innsbruck, Austria; 18 Department of Chemistry, University of Wisconsin-Madison, Madison, WI 53706, USA; 19 Institut IEK-8, Troposphäre, Forschungszentrum Jülich, 52425 Jülich, Germany; 20 Stony Brook University, School of Marine and Atmospheric Sciences, Stony Brook, NY, USA; 21 Max Planck Institut für Chemie, 55128 Mainz, Germany; 22 Department of Earth and Environmental Sciences, Indian Institute of Science Education and Research Mohali, India; 23 University of Northern Colorado, School of Biological Sciences, Greeley, CO 80639, USA; 24 University Bayreuth, AgroEcosystem Research, BAYCEER, 95447 Bayreuth, Germany; 25 Aerosol Dynamics Inc., Berkeley, CA, 94710, USA; 26 USTHB, University of Sciences and Technology Houari Boumediene, Faculty of Chemistry, Algiers, Algeria; 27 Centre de Développement des Energies Renouvelable, CDER, Algiers, Algeria

## Abstract

Despite the known biochemical production of a range of aromatic compounds by plants and the presence of benzenoids in floral scents, the emissions of only a few benzenoid compounds have been reported from the biosphere to the atmosphere. Here, using evidence from measurements at aircraft, ecosystem, tree, branch and leaf scales, with complementary isotopic labeling experiments, we show that vegetation (leaves, flowers, and phytoplankton) emits a wide variety of benzenoid compounds to the atmosphere at substantial rates. Controlled environment experiments show that plants are able to alter their metabolism to produce and release many benzenoids under stress conditions. The functions of these compounds remain unclear but may be related to chemical communication and protection against stress. We estimate the total global secondary organic aerosol potential from biogenic benzenoids to be similar to that from anthropogenic benzenoids (~10 Tg y^−1^), pointing to the importance of these natural emissions in atmospheric physics and chemistry.

Terrestrial vegetation is the largest source of reactive volatile organic compounds to the atmosphere, with hundreds of different compounds known to be produced and emitted by plants^1^,^2^,^3^,^4^. However, the emissions of only a few benzenoid compounds have been reported from plants^5^,^6^,^7^, despite biochemical evidence for the known production of a broad array of aromatic compounds by different metabolic pathways and the presence of benzenoids in floral scents^8^,^9^. Benzenoid compounds such as toluene, benzene and xylene are major components of oil and gasoline^10^ and are known to be emitted into the atmosphere when these fuels evaporate or are partially combusted. Their presence in the atmosphere is widely assumed to derive solely from these anthropogenic sources^11^ – the possibility that there may be significant biogenic sources of these compounds to the atmosphere has not previously been considered in regional and global trace gas emissions models or in modeled estimates of the occurrence of secondary organic aerosol in the atmosphere^12^,^13^.

Plants have developed a wide array of biochemical defense strategies to protect against biotic and abiotic stresses^14^,^15^, and one general response to stress in plants is the enhancement of secondary metabolism^16^. A number of different aromatic benzenoid compounds are rapidly produced in plants (and microorganisms) in response to stress, for example in the accumulation of isoflavonoids in response to ozone-stress^17^. A generic schematic showing the pathways that lead to the production of specific groups of volatiles, including benzenoids, is presented in Supplementary Fig. 1. Examples of biogenic benzenoids include herbivore-induced indole which attracts wasps which may protect plants^18^ and primes neighboring leaves to respond more strongly to subsequent herbivore attack^19^. Estragole is another example of a benzenoid emitted in response to attack (in this case by bark beetles)^20^, while salicylic acid is responsible for eliciting the expression of defense mechanisms, for example under pathogen attack or in disease resistance^20^. There are also other benzenoids which mediate plant interactions with insects or bacteria^21^. However, the biological functions of some benzenoid compounds, e.g. toluene, are still unknown. This lack of understanding mirrors the state of knowledge concerning many other important compounds of biogenic origin, including isoprene, two decades ago.

The shikimate pathway^21^ is an early step in the biochemical production of many, but not all, benzenoid compounds in plants. This crucial pathway is primarily devoted to the synthesis of aromatic amino acids (phenylalanine, tyrosine and tryptophan), which are the precursors for proteins and numerous natural products such as pigments, hormones, vitamins, alkaloids and cell-wall components^22^. Approximately 20% of the fixed carbon in plants flows through the shikimate pathway^23^. Under stress conditions, the requirements for the final products of the shikimate pathway (chorismate and isochorismate) may be enhanced due to activation of secondary metabolic routes, leading to the production of a variety of specific volatile benzenoid compounds for chemical signaling. In the case of wounding stress (e.g. by herbivores), more lignin, also derived via the shikimate pathway, may be required to rebuild cell walls^21^.

One common route for the formation of volatile benzenoids in plants starts from phenylalanine. In a reaction catalyzed by phenylalanine ammonia lyase, phenylalanine is converted to ammonia and cinnamate, a precursor for many benzenoid compounds including benzoic acid, an immediate precursor for salicylic acid^24^, although salicylic acid can also be synthesized directly from phenylalanine^25^ or via isochromate^26^. Cinnamate can also initiate the production of lignins, flavonoids, xanthones, phenolics, and other natural products^27^. Other benzenoids, including indole, are formed directly from chorismate, catalyzed by anthranilate synthase, the enzyme which converts chorismate to tryptophan^22^. The exact mechanisms behind the production and emission of a particular benzenoid may be quite complex, and indeed it is still unclear how toluene and benzene are formed in plants. Some non-volatile benzenoids can be enzymatically converted into more volatile derivatives which may then be emitted into the atmosphere. Hydroxylation, oxidation (to form benzaldehyde), methylation (of chavicol or salicylic acid), and acylation (to form benzyl acetate) are typical examples of such reactions^28^.

Despite the known occurrence of biochemical mechanisms for the synthesis of volatile aromatic compounds by plants, emissions of these compounds from the terrestrial biosphere have not previously been considered to make an important contribution to the total flux of reactive trace gases to the atmosphere. Aromatic compounds have been recognized to arise from the flowering parts of plants in scents, but their total emission rates have not been considered to be significant compared to those from anthropogenic sources. In fact, benzenoids previously detected in forest air have been attributed to either anthropogenic interferences or to artifact formation in the analytical sampling systems used^29^,^30^.

There is very little evidence in the literature for emissions of volatile aromatics from the biosphere. There are limited reports of the emissions of estragole (methyl chavicol)^31^,^32^ and p-cymene (an aromatic monoterpene)^33^,^34^ from various plant species, one report of toluene emissions from sunflower (*Helianthus annuus*) and pines (*Pinus* spp)^7^, and the suggestion of summertime biogenic toluene emissions from forests in the north-eastern U.S.^35^. Clearly detectable emission fluxes of toluene have been observed from an oil palm plantation canopy, but not from a contiguous natural rain forest in Malaysia^34^. Toluene was clearly detectable in the air at ground level in the plantation, but not in the rain forest, pointing to the fact that some tree species emit this compound and others do not^34^. Several aromatic compounds, including phenols and methyl salicylate, have been reported to be emitted from grey poplar (*Populus canescens*) under oxidative stress^35^, and several benzenoid compounds were found in branch enclosures from the creosote (*Larrea tridentate*) bush^6^.

In summary, there is a significant body of observational evidence that suggests that some plants produce benzenoids, but there has not previously been a systematic investigation of this phenomena across scales. Benzenoids generally have high potentials to produce both secondary organic aerosols^36^ and tropospheric ozone^37^ in the atmosphere, leading us to hypothesise that biogenic benzenoid compounds may play important roles in the chemistry and physics of the global atmosphere^31^,^32^.

Here, we show in controlled environment laboratory chamber studies that many benzenoid compounds are indeed emitted from leaves during stress (heat, herbivore attack, light-to-dark transition). We report benzenoid emissions measured during numerous separate field experiments conducted in a variety of ecosystems, spanning broad regional and vertical scales at the aircraft, canopy, tree, branch, and leaf levels and we show further evidence of marine emissions from data obtained from ship cruises and mesocosm enclosures. We show data from labeling experiments that confirms that plants do indeed directly and rapidly incorporate ^13^C from ^13^CO_2_ into toluene, xylene and phenol during their biosynthesis and that these labeled compounds are then emitted from the plant. We use the MEGAN 2.1^38^ biogenic VOC emissions model, which previously accounted for some, but not all, benzenoid emissions to estimate the amount of reactive carbon emitted as benzenoids by plants to the atmosphere (see Table 1). The approach used for estimating benzenoid emissions with MEGAN 2.1 is to assign an emission factor that represents an average level of stress. The intent is to establish the potential importance of these emissions in order to drive future research that can provide the observations required to develop and parameterize algorithms that can better represent emission response to stresses. Since these aromatic compounds have a high propensity to undergo chemical reactions in the gas phase that lead to condensable aerosol precursors^36^, atmospheric chemistry models should therefore account for this important source for secondary organic aerosol (SOA). Finally, we make a first-order estimate of the total global SOA formation potential from biogenic benzenoids of approximately 10 Tg y^−1^. This will likely increase in the future due to rising temperature and other changes to the global environment which will lead to a greater likelihood of plants suffering abiotic and biotic stresses.

## Results

### Controlled environment laboratory experiments

**Figure 1:**
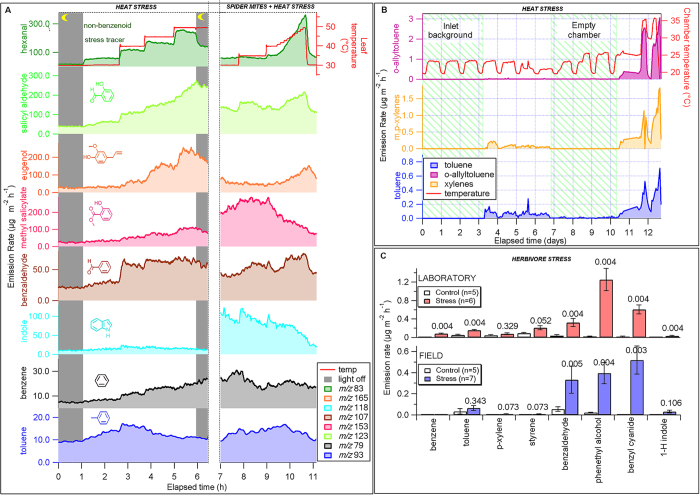
Laboratory studies reveal benzenoid compounds emitted from plants in response to stresses. **A**) Emission rates of seven benzenoid compounds emitted from *Populus balsamifera* in response to heat stress (left) and spider mite stress followed by heat stress (right). **B**) Heat-stress induced benzenoids from the Jülich controlled environment plant chamber containing three tree species (*Pinus sylvestris*, *Picea abies*, and *Betula*); **C**) Benzenoids from *Populus tremula* L. × *tremuloides* Michx. induced by larvae-feeding stress. Detailed descriptions of measurements can be found in the Supplementary Methods (S2).

In order to understand whether or not benzenoid emissions from plants are related to abiotic stress, we conducted a number of laboratory heat and herbivore stress experiments (Fig. 1; see also Supplementary Table 1 and Supplementary Fig. 3). Initial stress treatments of *Populus balsamifera*, which involved wounding, application of methyl jasmonate, fumigation with ethylene and fumigation with nitrogen oxide did not yield any significant benzenoid emissions, with the exception of a plant that was infested with spider mites. When plants were heat-stressed, however, we observed a wide range of aromatics released by leaves.

Heat stress treatments were performed under both light and dark conditions, including: 1) gradual temperature ramps; 2) fast temperature ramps and; 3) short term high temperature exposure (Fig. 1). Emissions of eugenol (*m/z* *+* 165) and salicylic aldehyde (*m/z* *+* 123) were induced by temperature-stress and increased significantly above 40 °C. A similar but less pronounced behavior was observed for benzaldehyde (*m/z* *+* 107). Emissions from *Populus balsamifera* infested with spider mites were also observed using an enclosure (right hand side of Fig.1A) and similar trends were observed for eugenol, benzaldehyde and salicylic aldehyde but with much higher emissions of indole (*m/z* *+* 118) and methyl salicylate (*m/z* *+* 153) than from the non-infested plants. The emission rates of both indole and methyl salicylate decreased with temperature, but emission rates of eugenol, benzaldehyde, and salicylic aldehyde increased with temperature, as from the non-infested controls.

Interestingly, the response of toluene to heat stress was different to that of eugenol or salicylic aldehyde. After toluene reached an emission maximum at around 35 °C, coincident with maximum photosynthesis, emissions declined with further increasing temperature. Toluene emissions are therefore likely tightly linked to photosynthesis (see Supplementary Figure 2). During rapid temperature increases the shutdown of photosynthesis may not be sufficiently fast to avoid large emission spikes of toluene in response to rapidly ramping the temperature to 50 °C. Such short exposure heat stress allowed for the full recovery of the leaf. A similar spike in temperature can occur in nature when there are sudden increases in incident solar radiation, especially in the tropics (e.g., sun flecks). Spiking temperature both at night and during the day triggered emission bursts of toluene which were not observed during gradual ramps. Such short-term bursts were sometimes encountered during the transition from dark to light, consistent with morning toluene releases observed in desert plant enclosures. Whether this is a communication signal or just the release of accumulated nocturnal metabolite is unknown. Exposure of *Populus balsamifera* to a leaf temperature of 55 °C was sufficient to cause permanent damage. However, the large benzenoid emissions observed at that temperature are probably due to the direct pyrolysis of tissue rather than heat stress.

In order to understand if benzenoid emissions occur only from flowers or also come from leaves, chamber experiments with enclosed plants were carried out. In these experiments, young trees, but not their roots or soil, were enclosed in controlled environment chambers, and it was confirmed that toluene, xylene and allyltoluene are indeed emitted from Scots pine (*Pinus sylvestris*), spruce (*Picea abies*) and silver birch (*Betula pendula*) under heat stress (Fig. 1). While biogenic source of VOC emissions are generally dominated by foliar and floral emissions, toluene emissions were also found from both air-dried and heat-treated *Pinus sylvestris* wood^39^, so bark, phloem and xylem of stem and branches of growing vegetation should be considered as a potential source of toluene.

Figure 1B also shows that emissions of benzenoids from *Populus tremula* L. × *tremuloides* Michx. are induced by larvae-feeding stress. This effect was observed both in controlled environment chamber studies and in the field using infested and non-infested leaves of the same tree species.

During a mesocosm experiment conducted to study VOC fluxes from phytoplankton, significant toluene emission fluxes to the atmosphere (mean = 0.5 μg m^−2^ h^−1^; max ~ 5 μg m^−2^ h^−1^) from seawater containing phytoplankton were observed. Remarkably, the flux correlated with the abundance of picophytoplankton and *Emiliania huxleyi* (the most dominant coccolithophore in the global ocean). As these emissions occurred after the peak in phytoplankton bloom, it is possible that the flux could have been much greater during the peak bloom, but this was not captured by the observations.

In order to confirm that benzenoid emissions are due to direct biosynthesis, controlled environment labeling experiments using ^13^CO_2_ were carried out using Scots pine (*Pinus sylvestris* L.), ponderosa pine (*Pinus ponderosa,* L.), silver birch (*Betula pendula* L.), poplar (*Populus x canescens*), English oak (*Quercus robur* L.), and tomato (*Lycopersicum esculentum*, cv. Moneymaker). The degree of ^13^C-labelling in the emissions of the benzenoids toluene, xylene, phenol and methyl salicyclate was then quantified under controlled light and temperature regimes. The degree of labeling varied from plant to plant and compound to compound. In some cases no labelling of VOCs was observed, in others more than half of the emitted aromatic molecules contained excess ^13^C, indicating the rapid assimilation of atmospheric carbon from CO_2_ into the emitted benzenoid compounds (see Supplementary Information for experimental details and data). Interestingly, all four of the target aromatic compounds were never fully labeled, indicating that there are either biosynthetic pathways for these compounds that do not use freshly incorporated CO_2_, or that the precursor pool is large and it would take longer than the length of the experiment for complete labeling to occur. This has also been previously observed in the case of isoprene biosynthesis in Sitka spuce (*Picea sitchensis*)^40^.

### Field observations

Having observed significant amounts of aromatic compounds, including toluene, benzene, benzaldehyde, salicylic aldehyde, eugenol, estragole and indole, being emitted by a wide range of plants in controlled environment laboratory experiments, we now look for evidence of biogenic benzenoid emissions from field observations.

**Figure 2:**
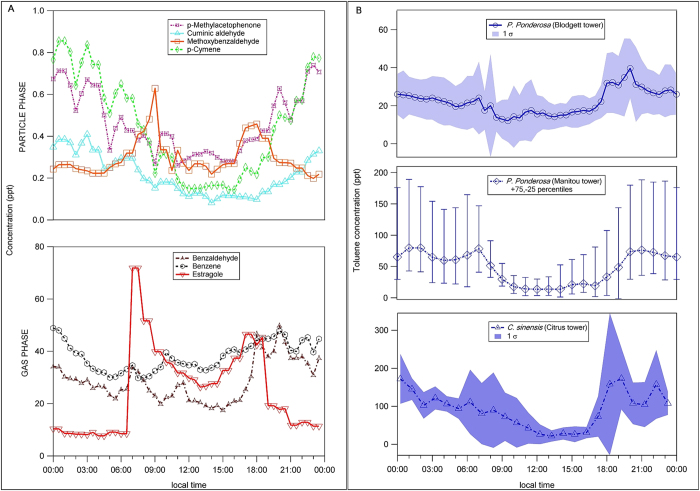
Field observations of concentrations point to the biogenic origin of benzenoid compounds. **A**) Gas-phase and particle phase observed benzenoids (other than toluene) at Blodgett forest during BEARPEX 2007; Methoxybenzaldehyde (particle) is oxidation product of estragole (gas). **B**) Concentrations of toluene above different vegetation canopies show consistent diurnal patterns with clear nocturnal accumulation when turbulence is low. The data were obtained with a range of analytical approaches, each of which is discussed in the Supplementary Methods (S2).

Figure 2A shows the concentrations of both gas-phase and particle-phase benzenoids made above a ponderosa pine canopy^41^. Factor analysis suggests that some of these compounds originate from a direct biogenic source or result from the oxidation of estragole (methyl chavicol). Two types of potential biogenic benzenoid source markers with different diurnal patterns are observed.

**Figure 3:**
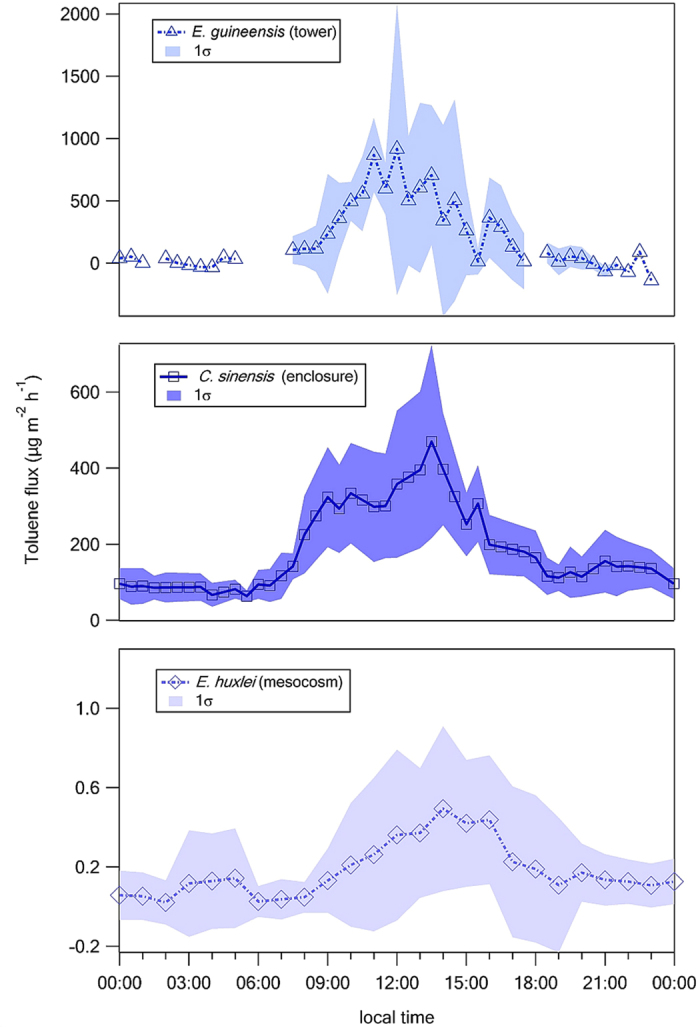
Field observations of fluxes of biogenic benzenoids show broad range of emissions. Fluxes of biogenic toluene (different species and scales). Large emission rates are typically observed during flowering and phytoplankton bloom. While toluene emissions from phytoplankton (*E. huxlei*) are an order of magnitude smaller than the emissions from flowering oil palm (*E. guineensis*) and citrus trees (*C. sinensis*), the global flux from phytoplankton is expected to be relatively high due to the larger area of oceans.

Figure 2B shows observations of diurnally varying concentrations of toluene above different vegetation canopies at a variety of field locations, with generally higher concentrations in the early morning and early evening and lower concentrations in the middle of the day. The expected atmospheric lifetime of toluene, based on its reactivity with the hydroxyl radical, is a few days, and hence the observed depletion of toluene in above-canopy air during the daytime is most likely due to enhanced dispersion into the boundary layer during the more turbulent daytime compared with nighttime. This is not necessarily indicative of enhanced emissions at nighttime. When we examine data from the direct measurement of toluene fluxes made over different vegetation canopies (Fig. 3), a strong diurnally-varying profile is observed, with maximum emissions occurring during the middle of the day and reduced or zero emissions at night time, consistent with our laboratory experiments.

Benzenoid emissions were also observed from ship-borne measurements over phytoplankton in the northern Atlantic, and four phytoplankton species, of the five selected for investigation in the laboratory, were subsequently found to be producers of toluene, although their culture densities were much higher than that encountered in nature.

Measurements made above two forest ecosystems in Colorado show that the concentrations of toluene declined with altitude, suggesting the presence of a source of toluene at the surface. The concentrations observed are similar to those of White *et al.*^33^ who hypothesized that forests may be a source of toluene to the atmosphere.

### Modelling global benzenoid emissions

**Figure 4:**
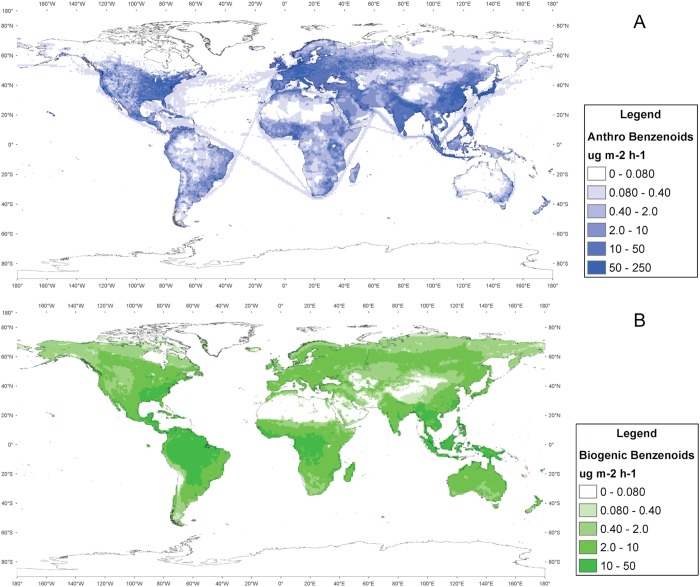
Spatial distributions (as annual average emissions) of anthropogenic (**A**) and biogenic (**B**) benzenoids. Biogenic benzenoid emissions are expected in remote areas where anthropogenic pollution (NOx) is lower so undergoes different oxidation and yields of SOA. Anthropogenic data were taken from RETRO 2000 and the biogenic distribution was calculated using MEGAN 2.1. Maps were created using ArcGIS software by ESRI (Environmental Systems Resource Institute, ArcMap 10.2, (www.esri.com)).

On the basis of our laboratory and field observations, we derive new benzenoid compound specific and ecosystem specific emission factors, shown in Table 1, for use in the MEGAN model^38^ of emissions of trace gases from nature. We then use MEGAN to estimate the global emission rates for these compounds, as shown in Fig. 4. Table 1 also shows possible drivers (floral, stress, etc.), the probable emission ranges for some of these compounds based on the present study, and their SOA formation estimates. We estimate total global biogenic emissions of toluene, benzene, xylene and other benzenoids to be in the range 4–40 Tg y^−1^, which compares with the current best estimate of anthropogenic benzenoid emissions of ~24 Tg y^−1^. From this, we estimate the global production rate of SOA from these biogenic benzenoid emissions to be 1.4–15 Tg y^−1^, compared with 2–12 Tg y^−1^ from anthropogenic benzenoid emissions.

## Discussion

Current global VOC emission inventories suggest that biogenic benzenoid emissions are much lower than those from anthropogenic sources, whereas the opposite is true for non-benzenoid biogenic VOC emissions. Indeed, some emission inventories disregard biogenic sources of benzenoids altogether. However, our experimental and field observational evidence clearly indicates that the biosphere is an important source of benzenoids to the atmosphere and that current inventories underestimate biogenic benzenoid emissions. Our global modelling suggests that the magnitude of biogenic emissions of these compounds may be comparable to anthropogenic emissions. Furthermore, the anthropogenic and biogenic benzenoid sources have different global spatial patterns (Fig. 4). SOA yields from these compounds are much higher under low NO_x_ conditions in the atmosphere compared with under polluted high NO_x_ conditions^36^.

Figure 4 shows that biogenic benzenoid emissions tend to occur in areas of the world with low NO_x_ conditions, while anthropogenic benzenoid emissions are co-located with anthropogenic sources of NO_x_ and hence with high NO_x_ conditions in the atmosphere. The magnitude of total SOA formation rates in the global atmosphere remains uncertain, although current estimates^1^ suggest the total global SOA source is at least 140 TgC/y. While 600 Tg/y of isoprene^2^ is likely to produce around 30 Tg/y SOA at a relatively low yield of 2%, aromatic compounds have very high aerosol yields^42^ of around 20% in low NO_x_ conditions^36^,^43^, as are typical for forested regions, especially at high and low latitudes. Hence, biogenic benzenoids are likely to produce about 10 Tg/y SOA, comparable with the SOA formation rate from anthropogenic benzenoids.

The emissions of benzenoids from plants may increase in the future owing to the rapid expansion of the cultivation of biofuel crops such as oil palm and maize and increasing phytoplankton biomass (our current estimate of biogenic benzenoid emissions from global oceans is around 10% of the total biogenic benzenoid emissions). Furthermore, different plants are able to synthesize specific benzenoids (e.g., for semiochemical and defense purposes), which are hard to detect at the ecosystem scale, but their cumulative contributions are probably important for SOA formation. These stress-induced emissions may increase in the future as global environmental change imposes increasing stresses on the biosphere.

## Additional Information

**How to cite this article**: Misztal, P.K. *et al.* Atmospheric benzenoid emissions from plants rival those from fossil fuels. *Sci. Rep.* **5**, 12064; doi: 10.1038/srep12064 (2015).

## Supplementary Material

Supplementary Information

## Figures and Tables

**Table 1:** Biogenic benzenoid volatile compounds.

**Compound**	**Biogenic driver**	**Biogenic (MEGAN v2.1)**		**Biogenic (possible range)**	**Anthropogenic (EDGAR)**	**Biogenic SOA (mostly low NOx)**	**Anthropogenic SOA (mostly high NOx)**
		**Emission factor**	**Global emission**	**Global emission**	**Global emission**	**Potential global source range**	**Potential global source**
		**μg m**^2^ **h**^−1^	**Tg y**^−1^	**Tg y**^−1^	**Tg y**^−1^	**Tg y**^−1^	**Tg y**^−1^
toluene	multiple	9	1.5	1 to 6^b^	7.6	0.3 to 3.0	0.6 to 4.5
benzene	multiple	0	0	0.1 to 1	6.1	0.04 to 0.4	0.5 to 3.3
xylene	multiple	0	0	0.1 to 0.5	5.2	0.04 to 0.2	0.4 to 2.2
other^a^		37	6.6	3 to 33	5.5	1.0 to 11	0.2 to 1.8
**Total**		**46**	**8.1**	**4 to 40**	**24.4**	**1.4 to 15**	**2.0 to 12**
a*other biogenic benzenoids*							
homosalate	sunscreen	8.4	2.0	1 to 10			
ethylhexenyl salate	sunscreen	4.2	0.98	0.5 to 5			
cymene <para->	foliar, floral	7.5	0.9	0.5 to 5			
cymene <ortho->	foliar, floral	4.5	0.54	0.2 to 3			
methyl salicylate	stress	9	1.5	0.3 to 3			
p-cymenene (dimethyl styrene)	unknown	0.3 to 0.6	0.05	<0.1 to 1			
estragole (methyl chavicol)	floral, fruit conifer	0.9 to 1.65	0.18	0.1 to 5			
indole	stress	0.6	0.1	<0.1 to 0.2			
benzaldehyde	stress	0.15	0.05	<0.1 to 0.2			
methyl benzoate	unknown	0.15	0.05	<0.1 to 0.1			
m-cymenene	unknown	0.3	0.04	<0.1 to 0.1			
phenylacetaldehyde	unknown	0.15	0.05	<0.1 to 0.1			
anisole	floral	0.15	0.05	<0.1 to 0.1			
benzyl acetate	floral	0.3	0.1	<0.1 to 0.2			
benzyl alcohol	floral	0.15	0.05	<0.1 to 0.1			
eugenol	stress	0	<0.02	<0.1 to 0.2			
cinnamic acid	stress, floral	0	<0.02	<0.1 to 0.1			
coniferyl alcohol	unknown	0	<0.02	<0.1 to 0.1			
chavicol	floral	0	<0.02	<0.1 to 0.1			
safrole	unknown	0	<0.02	<0.1 to 0.1			
ethyl cinnamate	unknown	0	<0.02	<0.1 to 0.1			
salicylic aldehyde	stress	0	<0.02	<0.1 to 0.2			

Compared are emission factors and global emission estimates used by MEGAN, and possible global ranges based on current understanding. In addition, comparison with anthropogenic benzenoid compounds is given.

^a^other biogenic benzenoids,

^b^includes emissions from picoplankton and *E. hux* of 1.6 Tg y^−1^.
