# Supplementary material for: Atmospheric benzenoid emissions from plants rival those from fossil fuels
Source: Sci Rep. 2015 Jul 13;5:12064. doi: 10.1038/srep12064 (PMC4499884; doi:10.1038/srep12064)
Supplement: Supplementary Information [file srep12064-s1.pdf]

SUPPLEMENTARY INFORMATION

Atmospheric benzenoid emissions from plants rival those from fossil fuels

PK Misztal[1,2*], CN Hewitt[3], J Wildt[4], JD Blande[5], ASD Eller[6,7], S Fares[1,8], DR Gentner[9,10], JB Gilman[6,11], M Graus[6,11,#], J Greenberg[2], AB Guenther[2,12,13], A Hansel[14], P Harley[2,15], M Huang[2], K Jardine[16], T Karl[17], L Kaser[2,14], FN Keutsch[18,#], A Kiendler-Scharr[19], E Kleist[4], BM Lerner[6,11], T Li[5], J Mak[20], AC Nölscher[21], R Schnitzhofer[14], V Sinha[22], B Thornton[23], C Warneke[6,11], F Wegener[24,#], C Werner[24,#], J Williams[21], DR Worton[1,25], N Yassaa[26,27] and AH Goldstein[1]

(1) University of California Berkeley, Environmental Science, Policy, and Management, Berkeley, CA 94720, USA.
(2) National Center for Atmospheric Research, Atmospheric Chemistry Division, Boulder, CO 80301, USA.
(3) Lancaster Environment Centre, Lancaster University, Lancaster LA1 4YQ, UK.
(4) Institut IBG-2, Phytosphäre, Forschungszentrum Jülich, 52425 Jülich, Germany.
(5) Department of Environmental Science, University of Eastern Finland, 70211 Kuopio, Finland.
(6) CIRES, University of Colorado, Boulder CO 80309 USA.
(7) University of Colorado, Department of Ecology and Evolutionary Biology, Boulder, Colorado 80309 USA.
(8) Council for Agricultural Research and Economics, Research Centre for the Soil-Plant System, Rome, Italy.
(9) University of California Berkeley, Department of Civil and Environmental Engineering, Berkeley, CA 94720, USA.
(10) Yale University, Chemical and Environmental Engineering, New Haven, CT 06520, USA.
(11) ESRL-NOAA, Chemical Sciences Division, Boulder CO 80305 USA.
(12) Pacific Northwest National Laboratory, Atmospheric Sciences and Global Change Division, Richland, WA, USA.
(13) Washington State University, Department of Civil and Environmental Engineering, Pullman, WA, USA.

(14) University of Innsbruck, Institute for Ion Physics and Applied Physics, 6020 Innsbruck, Austria.

(15) Estonian University of Life Sciences, Department of Plant Physiology, Tartu, Estonia.

(16) Lawrence Berkeley National Laboratory, Climate Sciences Department, Berkeley, CA 94720, USA.

(17) University of Innsbruck, Institute of Atmospheric And Cryospheric Sciences, 6020 Innsbruck, Austria.

(18) Department of Chemistry, University of Wisconsin-Madison, Madison, WI 53706, USA.

(19) Institut IEK-8, Troposphäre, Forschungszentrum Jülich, 52425 Jülich, Germany.

(20) Stony Brook University, School of Marine and Atmospheric Sciences, Stony Brook, NY, USA.

(21) Max Planck Institut für Chemie, 55128 Mainz, Germany.

(22) Department of Earth and Environmental Sciences, Indian Institute of Science Education and Research Mohali, India.

(23) University of Northern Colorado, School of Biological Sciences, Greeley, CO 80639, USA.

(24) University Bayreuth, AgroEcosystem Research, BAYCEER, 95447 Bayreuth, Germany.

(25) Aerosol Dynamics Inc., Berkeley, CA, 94710, USA.

(26) USTHB, University of Sciences and Technology Houari Boumediene, Faculty of Chemistry, Algiers, Algeria.

(27) Centre de Développement des Energies Renouvelable, CDER, Algiers, Algeria.

#Present addresses: University of Innsbruck, Institute of Atmospheric and Cryospheric Sciences, 6020 Innsbruck, Austria (M.G.); Harvard University, Department of Chemistry and Chemical Biology, Cambridge, MA 02138, USA (F.N.K.); Universität Freiburg, Ecosystem physiology, 79110 Freiburg, Germany (C. Werner, F.W.)

Correspondence to Pawel K Misztal[1] Correspondence and requests for materials should be addressed to P.K.M. (Email: pkm@berkeley.edu).

Contents

1. SUPPLEMENTARY DISCUSSION 4

1.1 BIOCHEMICAL FORMATION OF BIOGENIC BENZENOIDS 4

1.1 ATMOSPHERIC BIOGENIC BENZENOID COMPOUNDS 5

1.2 TOLUENE EMISSION LINKED TO PHOTOSYNTHESIS 6

2. SUPPLEMENTARY METHODS **8**

2.1 SUMMARY OF EXPERIMENTS 8

2.2 LABORATORY MEASUREMENTS 9

2.2.1 Heat stress study at NCAR (Balsam poplar leaves) *10*

2.2.1.1 Leaf enclosure system 10

2.2.1.2 PTR-ToF-MS 11

2.2.1.3 PTR-MS 12

2.2.1.4 GC-MS 13

2.2.2 Heat stress study in the Jülich plant chamber (three tree species) *14*

2.2.3 Herbivore stress (Aspen seedlings) *14*

2.2.3.1 Experiment description 14

2.2.3.2 VOC collection and analysis 15

2.2.3.3 Statistical analysis 15

2.2.4 Light-to-dark transition stress study (Halimium) *15*

2.3 FIELD MEASUREMENTS 15

2.3.1 Canopy scale *16*

2.3.1.1 Ponderosa pine (Pinus ponderosa: Blodgett forest, California) 16

2.3.1.2 Ponderosa pine (Pinus ponderosa: Manitou forest, Colorado) 16

2.3.1.3 Oil palm (Elaeis guineensis: Borneo) 17

2.3.1.4 Orange trees (Citrus × sinensis: California) 17

2.3.2 Aircraft scale *17*

2.3.2.1 Flights over ponderosa pine forests in Colorado 17

2.3.3 Marine emissions (phytoplankton) *18*

2.3.3.1 Mesocosm enclosure studies of phytoplankton emissions 18

2.3.3.1 Cruise over phytoplankton 19

2.3.4 Branch and leaf enclosures *22*

2.3.4.1 Corn (Zea mays subsp. Mays: leaf enclosures) 23

2.3.4.2 Citrus plants 23

2.3.4.3 Desert plants 23

2.4 $^{13}CO_2$ LABELING EXPERIMENTS 23

2.4.1 Experimental *23*

2.4.2 Labeling of aromatic BVOC *25*

1. Supplementary discussion

1.1 Biochemical formation of biogenic benzenoids

Benzenoids are thought to be formed in plants through the shikimate pathway. There are a few exceptions to this (e.g. p-cymene from conversion of monoterpenes formed in the terpenoid pathway[1]). Currently, the pathway for toluene biosynthesis is unknown but isotopic studies suggested it is formed by plants beyond the shikimate pathway. Benzene bursts observed together with green leaf volatiles from *Halimium* (see Figure 1D) from a ^{13}C-position specific pyruvate labelling experiment suggest it might possibly be formed from the lipoxygenase pathway.

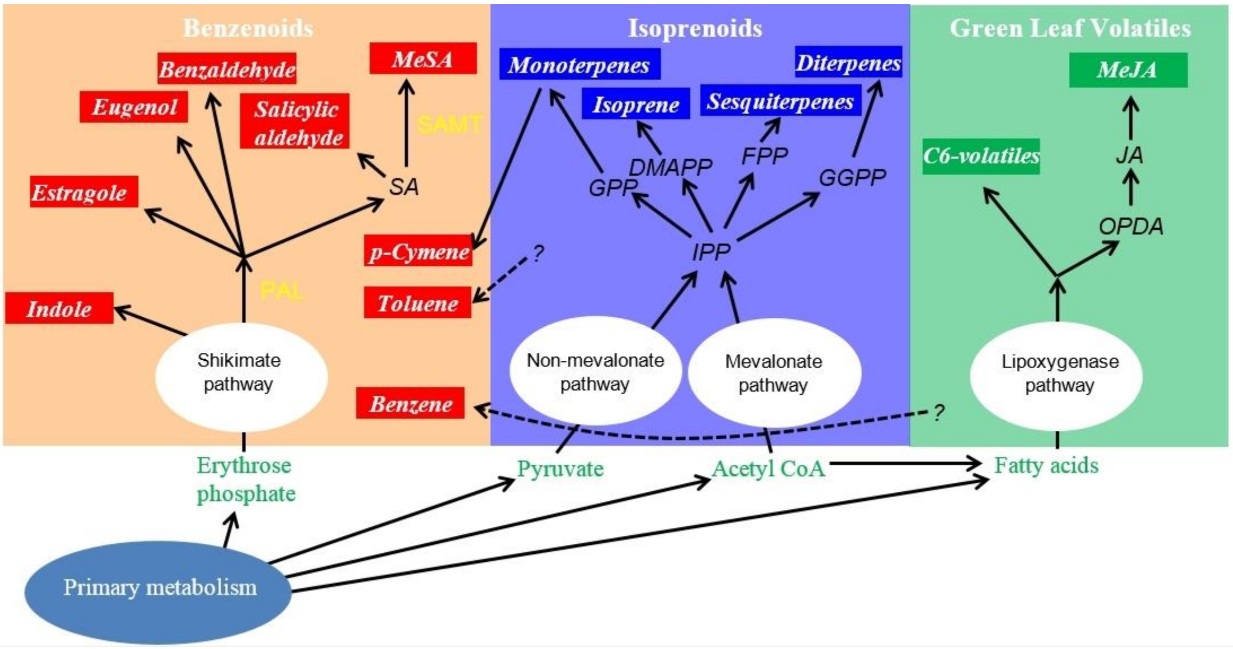

Supplementary Figure 1. *Generic pathways to which carbon can be diverted from the primary metabolism depending on the stress type and plant species. Pathways partially adapted from Kant et al.*[2] *and supplemented by current work. Acronyms: PAL L-phenylalanine ammonia-lyase; SAMT salicylic acid methyl transferase; SA salicylic acid; MeSA methyl salicylate; GPP geranyldiphosphate; GGPP geranylgeranyldiphosphate; IPP isopentenyl pyrophosphate; DMAPP dimethylallyl pyrophosphate; FPP C15-farnesyldiphosphate; JA jasmonic acid; MeJA*

methyl jasmonate; OPDA oxophytodienoic acid; ? – indicates unknown substrate and dashed line a hypothetical direction.

1.1 Atmospheric biogenic benzenoid compounds

Biogenic benzenoid release into the atmosphere can be divided into four main categories: 1) floral benzenoid emissions, which are relatively well known; 2) stress benzenoid emissions, which have been observed but are not well understood; 3) sunscreen compounds covering leaf surfaces for UV protection and 4) other possible benzenoid emissions.

To date, biogenic benzenoid emissions have not been regarded as important for atmospheric chemistry. Modeling of benzenoid emissions remains difficult because of the complexity of their primary and secondary metabolism, a lack of general understanding of plant responses to stress and the challenge of simulating ecosystem stress levels. Genetic explorations in combination with more targeted ecosystem studies employing new analytical instrumentation are needed to improve our understanding of the global magnitudes of aromatic compound emissions from vegetation. As in the case of aromatic semiochemicals (i.e. signaling chemicals), such as estragole and indole which modulate insect-plant interactions, we suspect many other benzenoid compounds which have not yet been detected could participate in chemical communication with other plants or insects. Plants have evolved the ability to generate chemical signals for their benefit. For example, floral mimicry can include imitations of rewarding flowers, egg deposition substrates such as rotting meat, feces, rotting meat or sexual signals[3]. However, it is currently not known how anthropogenic benzenoid compounds may interact with plant chemical communication in the short and longer term and whether pollution can increase or decrease biogenic benzenoid emissions.

1.2 Toluene emission linked to photosynthesis

The example data shown in Supplementary Figure 2 indicates that toluene synthesis and emission may be linked to photosynthesis. As the heat stress above 35 °C suppresses photosynthesis, toluene emission decreases. This is in contrast to other benzenoids which are thought to form starting from the shikimate pathway, which further increase with temperature stress (see Figure 1A). The shikimate pathway is the link between the primary and secondary metabolic pathway. Therefore those products (incl. chorismate and isochorismate) are also linked to photosynthesis. However, the plant can accumulate some products e.g. phenylalanine from the phenylpropanoid pathway and therefore some products will not become fully labelled if the plant is fed with $^{13}CO_2$. Toluene bursts following dark-to-light transition suggest that toluene can also be formed at night. Subsequent experiments showed that the morning bursts were only observed after the plant has been heat-stressed at night which may explain morning bursts observed previously in desert plants[4].

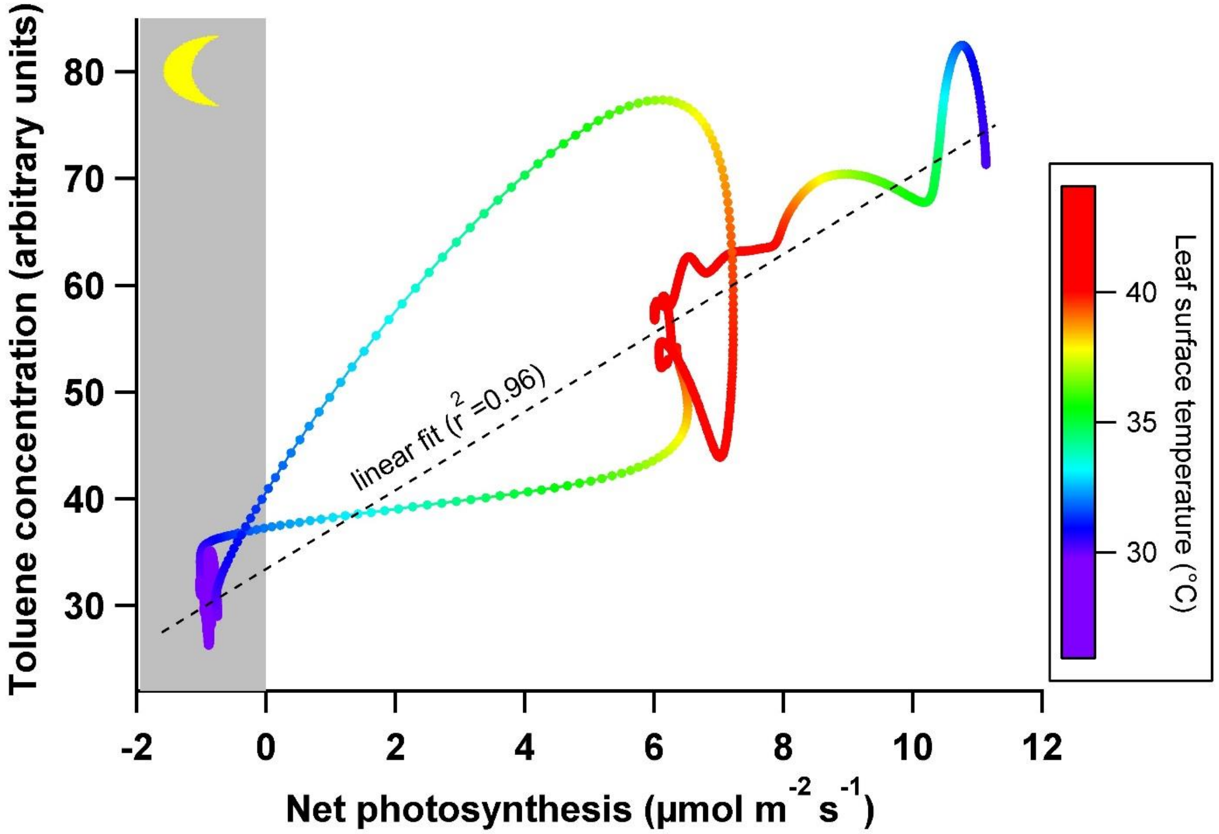

Supplementary Figure 2. *Toluene emission might be linked to photosynthesis. The highest toluene concentration was observed during the day at high net photosynthesis rates and when the leaf surface temperature reached an optimum value of typically 35 °C. A further temperature increase suppressed photosynthesis as well as the toluene emission.*

2. Supplementary Methods

2.1 Summary of experiments

Supplementary Table 1. *Summary of measurement techniques used in each study.*

Experiment	Analytical technique(s)	Calibration standard	Reference to experiment methods	Scale	Plant species
		LABORATORY OBSERVATIONS			
NCAR temperature stress 2011	PTR-TOF-MS	Y	Capellin et al.[5]	Leaf	*Populus balsamifera*
	PTR-Q-MS	Y	Fall et al.[6]		
	GC-MS	Y	Harley et al.[7]		
Jülich plant chamber temperature stress study	GC-MS	Y	Kleist et al.[8]	Tree	*Pinus sylvestris, Picea abies, Betula pendula*
Herbivore stress	GC-MS	Y	Li et al.[9]	Seedling	*Populus tremula* L. × *tremuloides* Michx.
Light-to-dark transition	GC-MS<br>PTR-QMS	Y	Jardine et al.[10]	Plant enclosure	*Halimium halimifolium* L.

FIELD OBSERVATIONS					
BEARPEX 2007	GC-MS<br>TAG-MS	Y<br>Y	Worton et al.[11]	Ecosystem	*Pinus ponderosa*
BEACHON-ROCS 2010	PTR-ToF-MS	Y	Kaser et al.[12]	Ecosystem	*Pinus ponderosa*
OP3 Oil palm	PTR-Q-MS<br>GC-MS	Y<br>Y	Misztal et al.[13]	Ecosystem, flower enclosure	*Elaeis guineensis*
Citrus campaign CA	PTR-ToF-MS<br>PTR-Q-MS<br>GC-MS	Y<br>Y<br>Y	Park et al.[14]	Ecosystem	*Citrus sinensis*
LongEZ flight over Manitou forest	PTR-MS	Y	Mak et al.[15]	Aircraft	*Pinus ponderosa*
Cruise over the northern Atlantic	GC-MS	Y	Yassaa et al.[16, 17]	Ship cruise	Phytoplankton (multiple species)

2.2 Laboratory measurements

2.2.1 Heat stress study at NCAR (Balsam poplar leaves)

2.2.1.1 Leaf enclosure system

The leaf enclosure system incorporated a dynamic, flow-through leaf gas exchange system (MPH-1000, Campbell Scientific, Logan, UT, USA) with a custom-built, temperature-controlled glass cuvette (of volume 400 cm^3). A balsam poplar (*Populus balsamifera*) leaf was inserted into the leaf cuvette and a controlled flow of humidified ultra clean air (produced by passing ambient air through a platinum catalyst heated to 450 ºC) enriched with CO_2 to approx. 400 ppm was passed through the cuvette. Artificial light was supplied by a metal halide lamp. Concentrations of VOCs in air exiting the cuvette were determined by a variety of analytical techniques (Supplementary Figure 3). Background VOC concentrations were obtained by making measurements in an empty cuvette for subsequent subtraction from leaf measurements.

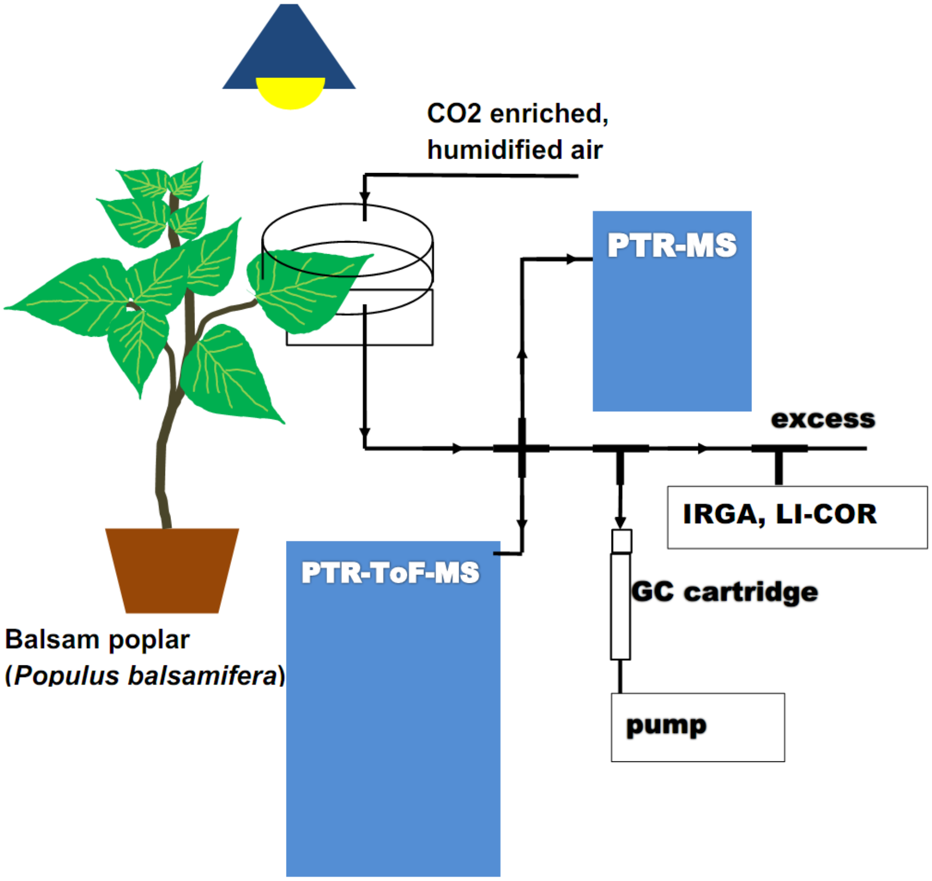

Supplementary Figure 3. *Schematic showing a simplified set-up of the laboratory experiment. The figure has been drawn using Microsoft PowerPoint 2013.*

2.2.1.2 PTR-ToF-MS

A commercial High Resolution Time-of-Flight Proton Transfer Reaction Mass Spectrometer (Ionicon PTR-ToF-MS 8000) instrument subsampled the air flowing from the leaf cuvette and measured full mass spectra up to 320 amu with high time resolution, offering exact mass determination of ionized VOCs. The instrument operation has been described by Graus et al.[18]. Confirmation by GC-MS suggests that in-situ monitoring of specific volatile aromatics such as eugenol, indole, salicylic aldehyde and benzaldehyde is feasible using the PTR-ToF-MS methodology. For identification of benzenoid compounds using precise *m/z*+ from PTR-ToF-MS and GC-MS, see Supplementary Table 2.

Supplementary Table 2. *Summary of biogenic benzenoid identification using PTR-ToF-MS and GC-MS for the laboratory Populus balsamifera heat-stress experiment. The unidentified peaks are presented in red.*

**Exact *m/z*+ [Th]**	**Measured *m/z*+ [Th]**	**Mass deviation [ppm]**	**Molecular Formula**	**Compound**	**Nominal-mass Percentage**	**GC-MS**
69.0699	69.0680	27.2	$C_5H_8H^+$	isoprene	100	Yes
79.0542	79.0520	28.2	$C_6H_6H^+$	benzene	100	Yes
93.0335	93.0340	5.5	$C_6H_4OH^+$	n/a	43	No
93.0699	93.0670	30.9	$C_7H_8H^+$	toluene	57	Yes
107.0491	107.0470	20.0	$C_7H_6OH^+$	benzaldehyde	76	Yes
107.0855	107.0830	23.6	$C_8H_{10}H^+$	xylene/ethylbenzene	24	Yes
118.0651	118.0623	23.9	$C_8H_7NH^+$	indole	100	Yes
123.0441	123.0430	8.6	$C_7H_6O_2H^+$	salicylic aldehyde	83	Yes
123.1168	123.1104	52	$C_9H_{14}H^+$	n/a	17	No
153.0546	153.0520	17.1	$C_8H_8O_3H^+$	methyl salicylate	82	Yes
153.1274	153.1200	52.2	$C_{10}H_{16}OH^+$	n/a	18	No
165. 0910	165.0880	18.2	$C_{10}H_{12}O_2H^+$	eugenol	100	Yes

PTR-ToF-MS allows for in-situ monitoring of full mass spectra with high time and mass resolution. Isobaric species having the same nominal mass but differing in atomic composition can be separated by the very high mass resolution of the PTR-ToF-MS instrument. Supplementary Figure 4 shows an example how PTR-ToF-MS can separate isobaric species at a nominal mass *m/z*+ 107.

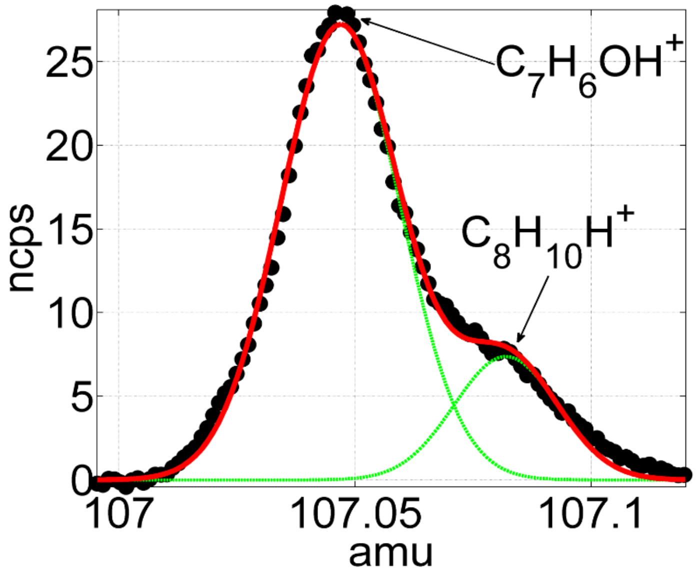

Supplementary Figure 4. *A multipeak fit for nominal mass m/z+ 107 using PTR-ToF-MS data (black dots). Green lines depict the relative contribution of each individual compound using a Gaussian peak form. The red line is the sum of the individual fit functions used to reconstruct the original signal. The dominant peak identified via isotopic mass ratios as* $C_7H_6OH^+$ *is consistent with benzaldehyde.*

2.2.1.3 PTR-MS

A high sensitivity quadrupole Proton-Transfer-Reaction Mass Spectrometer (Ionicon PTR-MS) sampled the outflowing air from the cuvette through 1/8'' PTFE tubing connected to a 4-way stainless steel junction. This flow was shared with the PTR-ToF-MS and the IRGA. The principles of operation of this particular instrument are presented elsewhere[19]. The flow to the PTR-MS was maintained at 150 $cm^3\ min^{-1}$. Drift-tube conditions were optimized for *E/N* of 118 Td (*E* being the electric field strength and *N* the gas number density; 1 Td=10^{-17} V cm^{-2}) which

was achieved by maintaining the drift pressure, drift voltage and drift temperature at 0.023 Pa, 565 V and 40 ºC, respectively. The acquisition was made by Ionicon LabVIEW Control software (beta version 2.7.42) which communicated to a custom built LabVIEW program through API features, displaying instantaneously normalized count rates. A 10 period running-average filter was applied to m/z+ 21 to enhance the signal to noise ratio, when normalizing ion signals to the H_3O^+ ion. During post-processing the normalized counts were subjected to normalized background subtraction and were divided by normalized sensitivities to yield concentrations, subsequently converted to emission rates.

The background atmosphere in the cuvette was measured in the same light and temperature regimes and was subtracted from all leaf measurements. In order to exclude any possible contributions from heated materials in the leaf cuvette the leaf was removed and the temperature was ramped in 5 ºC steps from 20 ºC to 50 ºC (and to 55 ºC on one occasion) with approximately 0.5 h hold at each temperature. In addition the effect of light was tested at low and high temperatures. Median values of the background at each temperature bin were subtracted from each measurement at the corresponding temperature ranges. Most of the ions seemed unaffected by temperature in an empty cuvette. However, *m/z*+ 95 was highly dependent on the temperature, exhibiting an exponential increase. To a much lesser degree, the same dependence was also noted for *m/z*+ 45, 59, 113, and 118. In the case of *m/z*+ 95 (typically attributed to phenol), which tended to be elevated in the leaf measurement, the subtraction of the backgrounds sometimes led to negative values and therefore attribution of this signal to biogenic origin was treated with caution and not included in the analysis.

2.2.1.4 GC-MS

The composition of the air exiting the cuvette was also determined using GC-MS. Air exiting the cuvette was pulled through two-stage adsorbent cartridges (175 mg Tenax GR 35/60 and 175 mg Carbograph 5TD 40/60; Markes Intl., Llantrisant, UK) at a flow-controlled rate of 196 sccm for 30 minutes (vol=5880 standard cm^3). Cartridges were thermally desorbed (Unity thermal desorber, Markes), and the samples separated on a DB-5 chromatographic column and analyzed by GC-MS (Model 7890A with Model 5975C Mass Detector, Agilent Technologies, Santa Clara,

CA). Compound identifications were based on comparison of mass spectra with those of the NIST mass spectrum library, and comparison with published retention times obtained using a DB-5 chromatographic column [20].

2.2.2 Heat stress study in the Jülich plant chamber (three tree species)

Experiments were conducted in an 1150 L plant chamber facility at Forschungszentrum Jülich, Germany (Jülich Plant Aerosol Atmosphere Chamber, JPAC). Three plant species (spruce, birch, Scots pine) were enclosed and monitored by a GC-MS for volatile organic compounds at controlled temperature and light conditions in the chamber. The operation of this particular chamber was described elsewhere[21]. Details of the setup are as described by Mentel et al.[22].

2.2.3 Herbivore stress (Aspen seedlings)

2.2.3.1 Experiment description

Both laboratory and field experiments were performed with 18 month old micro-propagated saplings of hybrid aspen (*Populus tremula* L. × *tremuloides* Michx.) with two-to-four branches. In the laboratory experiment, twelve healthy potted plants that were grown in the greenhouse were transferred to controlled environment chambers (Weiss Bio 1300; Weiss Umwelttechnik Gmbh, Preskirchen-Lindenstruth, Germany) and acclimatized for two days. Plants were then randomly assigned to either insect damage stress or control groups, and placed in separate chambers. In the insect damage treatment, 24 *Phyllodecta laticollis* larvae were placed on one upper branch of each plant, and allowed to feed on the branch for three days prior to VOC collection.

In the field experiment, aspen seedlings were transplanted in the Ruohoniemi experimental field site at the Research Garden of University of Eastern Finland in June 2011. In July 2012, seven plants without visible damage were infested with 25 *P. laticollis* larvae by applying them to the upper branch, while matching controls were not infested. After three days,

VOCs were collected concurrently from the damaged branches and the corresponding branches of the control plants.

2.2.3.2 VOC collection and analysis

Branches were enclosed in polyethylene terephthalate (PET) bags (Look; Terinex Ltd., Bedford, UK) that do not themselves emit detectable amounts of volatiles. The emitted VOCs were collected for 30 min on Tenax TA adsorbents (250 mg, mesh 60/80, Markes International, Ltd., UK) using air circulation as described previously (Blande *et al*. 2010). The VOC samples were analyzed by gas chromatography-mass spectrometry (Agilent GC 7890A, VL-MSD 5975C) coupled with a thermal desorption unit (TD-100, Markes International, Ltd., UK)[23]. All compounds reported here were identified by comparing their mass spectra with the Wiley library.

2.2.3.3 Statistical analysis

The Mann-Whitney U test was used to compare the differences between control and stress plants. All statistical analyses were performed using the software package SPSS statistics 16.0 (SPSS Inc., Chicago, IL, USA).

2.2.4 Light-to-dark transition stress study (*Halimium*)

The Mediterranean shrub *Halimium halimifolium* L. was potted in sandy soils and enclosed in glass chambers of volume 300 mL. The chambers were continuously flushed with 400 ml/min hydrocarbon free air and the chamber exposed to photosynthestically active radiation of 400 µmol m^{-2} s^{-1} at the top of the chambers.

2.3 Field measurements

Combined measurements of trace gases in the field (as shown in Figure 2) were performed using a wide array of state-of-the-art analytical approaches, including Proton Transfer

Reaction Mass Spectrometry (PTRMS), Proton Transfer Reaction Time of Flight Mass Spectrometry (PTR-ToF), Gas Chromatography – Mass Spectrometry (GC-MS), Gas Chromatography with Flame Ionization Detector (GC-FID), and Thermal Desorption Aerosol GC/MS (TAG). These methods provided concentration data and, where possible, fluxes by direct eddy covariance.

2.3.1 Canopy scale

2.3.1.1 Ponderosa pine (Pinus ponderosa: Blodgett forest, California)

There is a long-term record of tower-based BVOC emissions measurements from a homogenous ponderosa pine ecosystem at Blodgett Forest. This site has a well characterized meteorology. We use the benzenoid data from the BEARPEX 2007 campaign. Measurements were performed using an in-situ GC-MS for gas phase analytes and a Thermal desorption Aerosol GC-MS (TAG) for semivolatile and particle phase analytes. The methods used have been described elsewhere[24,11].

2.3.1.2 Ponderosa pine (Pinus ponderosa: Manitou forest, Colorado)

Ponderosa pine emissions have also been monitored from Manitou forest using a tower-based sampling system. Since benzenoid emissions from ponderosa pine are thought to be driven by biotic or abiotic stress, comparison of emission rates from different ponderosa pine ecosystems is helpful. The evidence presented here was obtained during the BEACHON-ROCS 2010 campaign using PTR-ToF-MS to measure fluxes and concentrations. Methods for flux derivation from PTR-ToF-MS have been described elsewhere[25,12]. Potential interferences with benzenoid compounds in PTR-MS and PTR-ToF-MS studies of this manuscript were carefully excluded using approaches described by Kaser et al.[12] and/or using verification with GC-MS. For example, the signal attributed to C_6H_6-H^+ protonated benzene (*m/z*$^+$ 79.055) may be due to the presence of a protonated acetic acid water cluster (*m/z*+ 79.039). Toluene is detected at *m/z*+ 93(.0699) by

PTR-MS. Using data from the literature[26] it is known that at an *E/N* of 124 Td about 67-71% of the cymene signal is found at *m/z*+ 93.0699, which interferes with toluene. After recalibration of the fragmentation pattern of cymene in the laboratory we used the measured *m/z*+ 135.117 signal to correct the *m/z*+ 93.0699 signal measured in ambient air at BEACHON-ROCS.

2.3.1.3 Oil palm (Elaeis guineensis: Borneo)

Toluene and estragole identification was obtained through the combination of PTR-MS and in-situ GC-MS data from a tower above an oil palm canopy and confirmed by flower enclosure studies[13, 27].

2.3.1.4 Orange trees (Citrus × sinensis: California)

Emissions of toluene were identified using an in-situ GC-MS. PTR-MS and PTR-ToF-MS instruments at the same site provided consistent results. Here only GC-MS data are shown. The anthropogenic influences from fossil fuels have been subtracted using the toluene to isooctane (gasoline tracer) ratio, which was derived from a tunnel study in California[28]. This approach improved the correlation of toluene with limonene (a non-benzenoid biogenic tracer).

2.3.2 Aircraft scale

2.3.2.1 Flights over ponderosa pine forests in Colorado

The experimental aircraft Long EZ N282RD flew profiles above two ponderosa pine ecosystems at Manitou forest and Black Forest, both near Colorado Springs, CO. The air was sampled using the Whole Air Sample Profiler (WASP) and subsequently analyzed in the laboratory using PTR-MS. Here, example data from two flights were selected (Jul 15, 2011 over Black Forest and Jul 20, 2011 over Manitou Forest).

WASP is an aircraft based system (Supplementary Figure 5). It is comprised of a long coiled tube (e.g. Silcosteel, 70 m in length, 1.25 cm diameter) into which sample air is flowed. The air sample is collected during flight, and returned to the laboratory for subsequent analysis. Sample

collection time is dependent upon air flow and tube length. An automated shutoff valve is operated by the pilot to begin and end sample collection.

Initial and final sample times are recorded, as are altitude, temperature, and relative humidity. In order to determine the precise position of the aircraft during sample collection, a pulsed tracer system is used. This system injects a discrete amount of appropriate tracer gas (e.g., SF_6 or C_4H_5N) at regular intervals. This pulsed injection system also allows for quantifying the rate of molecular diffusion that may take place during sample storage.

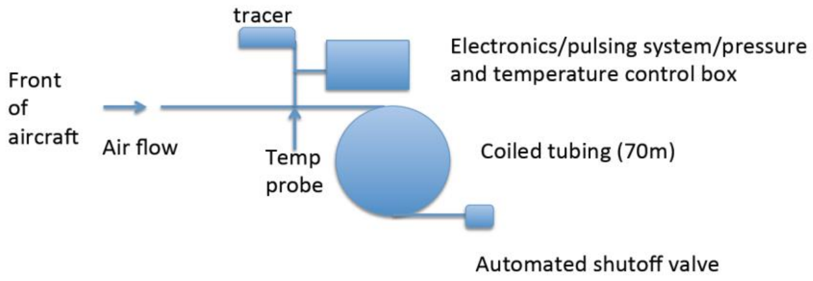

Supplementary Figure 5. *Schematic of WASP*

2.3.3 Marine emissions (phytoplankton)

2.3.3.1 Mesocosm enclosure studies of phytoplankton emissions

Sequential VOC measurements of ambient air and air from two duplicate mesocosms were made for 10 min each, in the order – mesocosm 1, ambient air, mesocosm 2, using a PTR-MS. The measurements were made in a period following a phytoplankton bloom and the chlorophyll and nutrient parameters were representative of vast areas of the global oceans. Details of the experimental set up and flux calculation are given elewhere[29].

2.3.3.1 Cruise over phytoplankton

Production rates of toluene from phytoplankton species were derived from samples taken during a ship cruise over the northern Atlantic. Details on the algae cultures, sampling procedure, blank tests and analysis of emitted compounds have been summarized in Yassaa et al.[17] and description of the Meteor Cruise 68/3, shipborne sampling, incubations etc. in Yassaa et al.[16]

<u>Incubation experiments during Meteor Cruise</u>

Description of the cruise Meteor 68/3:

The third leg of Meteor cruise 68 was carried out as part of the international “Surface Ocean Lower Atmosphere Study” (SOLAS). It combined a wide spectrum of biological, chemical and physical oceanography as well as atmospheric chemistry with a regional focus on Cape Verdean waters and coastal upwelling off Mauritania.

Upwelling regions in major dust deposition areas can be viewed as biogeochemical reactors which are fuelled simultaneously by vertical supply of macro- and micro-nutrients from the mesopelagic layer below and the atmosphere above. At the same time, these regions provide means of ventilation of radiatively and chemically active trace gases (e.g., CO_2, nitrous oxide, bromoform) which are produced sub-surface. The resulting flux densities are larger than in the oligotrophic background waters.

On-board instrumentation enabled key organic species to be measured in the atmosphere and ocean simultaneously. Between 12th July and 6th August the ship steamed from Mindelo/Cape Verde (16°N 25°W) to Las Palmas de Gran Canaria/Spain (28°N 14°W). This cruise track permitted measurements as a function of latitude, of longitude, time of day and for selected species as a function of depth.

Sampling procedure

During the Meteor Cruise, organic trace gases were measured from seawater through incubation experiments, as described in [16]. Duplicate 250 mL glass bottles (Schott, Germany)

fitted with Teflon caps (Bola, Germany) were constructed with three ports to which 1/4" Teflon tubing could be fixed and through which gas could be flushed for air sampling. A length of Teflon tubing connected a three port Teflon valve at the top of the bottle to below the liquid surface in the incubation flask, so that samples could periodically be drawn out without perturbing the experiment. Within each flask pair, one of the flasks was designated as a control and filled with filtered seawater (100 mL), while the second was filled with the investigated seawater. Filtered synthetic air was flushed continuously through both flasks at identical flow rates (100 mL min^{-1}). Air samples were then collected both from the air stream before entering the flasks for air blanks and also at the exit for the determination of the trace gas production of the various species. The difference between the inlet and outlet concentrations was attributed to the biogenic emission (or uptake) of the investigated species if the same difference was not observed in the control flask.

Air samples taken before and after the incubation flasks were collected through stainless steel two-bed sampling cartridges (Carbograph I/ Carbograph II; Markes International, Pontyclun, UK) at flow-rate of 100 mL min^{-1}. Prior to air sampling, the sampling cartridges were cleaned with the Thermoconditioner TC-020 (Markes International, Pontyclun, UK) by purging with helium 6.0 (99.9999%, Messer-Griesheim, Germany) for 120 min at 350°C and 30 min at 380°C. For storage the cartridges were sealed with brass caps with PTFE ferrules and put into an airtight metal container (Rotilabo, Carl Roth GmbH & Co, Karlsruhe, Germany). After sampling, the cartridges were stored in a separate airtight metal container. Shortly before analysis, the brass caps were exchanged for DiffLok-caps (Markes International, Pontyclun, UK).

<u>Laboratory incubation experiments</u>

Algae culture, sampling procedure, blank tests and analysis of emitted compounds are all detailed in our previous work[17]. Here we present only a brief description.

Algae culture and species

All emission analysis experiments were carried out with batch cultures of algae provided by IFM-GEOMAR, Kiel. Prior to the experiments, all cultures were kept at room temperature

between 20-25°C and adapted to a 12-12 hour light-dark cycle. The two coccolithophorids, *Calcidiscus leptoporus* (strain AC365, from South Atlantic off South Africa, CODENET culture collection, ALGOBANK <http://www.unicaen.fr/algobank) and *Emiliania huxleyi* CCMP 371, the two diatoms *Chaetoceros neogracilis* CCMP1318 and *Phaeodactylum tricornutum* (Phaeo, originating from the Falkowski laboratory) and the chlorophyte *Dunaliella tertiolecta* (DUN, originating from the Falkowski laboratory) were grown in f/2-medium. These last four cultures were kept axenic (bacteria free) prior to the experiment. These cultures are frequently used at IFM-GEOMAR Kiel for various purposes and checked regularly with "Marine Broth" for contamination of bacteria. So, *Calcidiscus leptoporus* was not axenic, but all CCMP1318 and CCMP 371 were also axenic according to the CCMP culture collection list and *Phaeodactylum tricornutum* and the chlorophyte *Dunaliella tertiolecta* are our home growth axenic cultures. All algae were in the transition from the exponential to the stationary phases of growth. This was measured using a PhytoPAM (WALZ).

The light intensity was approximately 250 µE s^{-1} m^{-2} and the samplings of VOC emissions were performed in the middle of the light cycle (between 10:00-14:00). Chlorophyll a was measured according to the following procedure: 10 mL of the cultures were filtered through GF/F filters and frozen at -20°C. Chlorophyll was extracted with 10 ml 90 %-acetone, centrifuge and the supernatant was measured fluorometricaly with a Turner fluorometer according to Welschmeyer.

Blank tests

Prior to the transfer of algae and control from Nalgene to the Duran bottles, blank tests were performed with empty bottles. Further controls were performed with bottles containing only the f/2 medium, which was used to cultivate the phytoplankton species. Most plastics are permeable to light hydrocarbons, and several have been shown to actively absorb these chemicals, and sometimes subsequently to re-emit them. For these reasons glass vessels were used in our experiments. The VOC mixing-ratios in the headspace above the f/2 medium were defined as "control". The emissions from phytoplankton were defined as occurring only when the headspace mixing ratio for a given benzenoid compound was higher than the control headspace mixing ratio.

Headspace solid phase microextraction (SPME)

Sampling procedure

First, 200 mL of algae suspension from each species was separately transferred from Nalgene incubation bottles into 250 mL Duran glass bottles fitted with a PTFE-septum. A further identical glass bottle was left empty as a blank. VOCs emitted from each algae species was then extracted by exposing a 65 µm polydimethylsiloxane/divinylbenzene (PDMS/DVB) fibre installed in an SPME holder for manual procedure (fibre and holder purchased from Supelco, Taufkirchen,Germany) to the headspace of each bottle for 30 minutes at ambient temperature and without pre-filtering or agitation, so that no modification in the growing conditions of algae took place.

Chromatographic analysis

The extracted VOCs in the SPME fibre were thermally desorbed (at 250°C) into a gas chromatograph injection port fitted with a SPME-liner (Supelco, Taufkirchen, Germany) for 5 min. For this purpose, a gas chromatograph (Agilent Technologies a GC 6890A) coupled to a Mass Selective Detector (MSD 5973 *inert*) from the same company was employed for qualitative and quantitative analysis. The MSD was operated in electron impact mode with the following conditions: ionization energy 70 eV; source temperature 230°C; and scan number (30-550 amu). A □-cylodextrin capillary column (CYCLODEX-B, 30 m-long, 0.256 mm ID, 0.25 µm film thickness) supplied by J & W Scientific (California, USA) was installed for the separation of enantiomeric monoterpenes and other VOCs. The initial column temperature was maintained at 40°C for 5 min, then increased to 200°C at 1.5°C/min and held at this temperature for 5 min.

2.3.4 Branch and leaf enclosures

2.3.4.1 Corn (Zea mays subsp. Mays: leaf enclosures)

Benzenoids from corn were measured during BioCORN 2011 and the data reported here come from enclosures analyzed by an online GC-MS (TACOH). The samples were taken using a LICOR Li-6400, which was the same as used in S2.2.1.1. Ambient PAR and temperature were maintained over a 40 h period using 4 different plants. Although benzene and toluene emissions were small, toluene showed higher emissions in the afternoon than at other times. Benzene emissions were noisier, but its emissions show a diurnal course similar to that of toluene, but at lower magnitude and with more scatter. P-cymene emissions were below the detection limit. Benzaldehyde was also emitted during the day in comparatively appreciable amounts. An earlier study[30] above corn also showed elevated concentration of some benzenoids but the enclosure study presented here confirms that at least part of those ambient concentrations have biogenic origin.

2.3.4.2 Citrus plants

Whole orange branches were enclosed in Teflon chambers and volatiles were monitored by PTR-MS. Methodology and results were described by Fares et al.[31]

2.3.4.3 Desert plants

The details of the branch enclosures used and results are described by Jardine et al.[4]

2.4 $^{13}CO_2$ labeling experiments

2.4.1 Experimental

$^{13}CO_2$ labeling experiments were carried out using a borosilicate glass chamber of volume 164 L. The chamber was operated as a continuously stirred tank reactor (CSTR) with a Teflon fan ensuring homogeneous mixing, with mixing times of about two minutes. Eight discharge lamps (HQI 400 W/D; Osram, Munich, Germany) were used to simulate the solar light

spectrum. At full illumination and at typical mid-canopy height photosynthetic photon flux density (PPFD) was 800 µmol·m^{-2}·s^{-1}. The glass chamber was housed in a walk-in climate chamber to ensure constant temperature. Teflon tubes were used as connections between the chamber and analytical equipment. Differences of CO_2 and H_2O mixing ratios between the chamber outlet and inlet were measured using commercial equipment (Binos, Rosemount). Mixing ratios of biogenic volatile organic compounds (BVOC) were determined by gas chromatography - mass spectrometry (Agilent GC-MSD-system HP5890 Series II + HP5972A MSD, GC-MSD-system HP6890 + 5973 MSD) equipped with thermal desorption systems (online TDSG, Gerstel, Mülheim, Germany). For more details on the systems used see e.g. Heiden et al.[21]. In one case a Proton Transfer Reaction Mass Spectrometer (PTR-MS, Ionicon, Innsbruck, Austria) was also used.

During experiments without $^{13}CO_2$ exposure, the chamber was flushed with air purified by an adsorption dryer (KEA 70; Zander Aufbereitungstechnik GmbH & Co. KG, Essen, Germany) and by a palladium catalyst operating at 450°C. Ozone, NO, NO_2, and volatile organic compounds (> C_3) were removed by the purification system. Since the concentrations of CO_2 were reduced by the air cleaning system, CO_2 (Linde, Air Liquide) was added to the chamber to reach experimental concentrations of around 350 ppm.

During $^{13}CO_2$ exposure experiments the chamber was flushed with CO_2 free synthetic air taken from cylinders (Linde, Air Liquide, 20.5% O_2 in N_2). $^{13}CO_2$ from another cylinder (99 % ^{13}C, Sigma Aldrich, Chemotrade) was added via a flow-controller (Brooks). The flow was adjusted to reach ~350 ppm $^{13}CO_2$ in the chamber. Typical air flows used during these experiments were between 5 and 10 L min^{-1} synthetic air.

A diurnal light cycle was simulated in the plant chamber using lamps (6:00 to 18:00 full illumination, 18:00 to 19:00 simulation of twilight by sequential reduction of illumination, 19:00 to 05:00 darkness, and 05:00 to 06:00 simulation of twilight by sequential increase of illumination). $^{13}CO_2$ addition was carried out during full illumination. Exposure durations were between 3 and 8 hours. In several cases the period of illumination was elongated after switching

back from 99% $^{13}CO_2$ to CO_2 with natural ^{13}C abundance in order to follow the decay of the degree of labeling.

Labeling experiments were conducted with Scots pine (*Pinus sylvestris* L.), ponderosa pine (*Pinus Ponderosa*, L.), silver birch (*Betula pendula* L.), poplar (*Populus x canescens*), English oak (*Quercus robur* L.), and tomato (*Lycopersicum esculentum*, cv. Moneymaker). The aim of the labeling experiments was to check if the respective BVOC was biosynthesized by the plant, as indicated by ^{13}C incorporation into the emitted BVOC. To demonstrate ^{13}C incorporation we used the ratio labeled over unlabeled BVOC, R_{iso}, (see e.g. Kleist et al.[32]):

$$R_{iso} = \frac{labeled}{unlabeled} = \frac{C(m+1)+C(m+2)+...C(m+n)}{C(m)} \qquad (1)$$

In Eq. (1), *C*(m) is the count rate obtained from the mass spectrum for the molecular ion. *C*(m+1) to *C*(m+n) are the count rates obtained at the masses m+1 to m+n with n being the number of *C* atoms contained in the respective BVOC molecule. Equation (1) gives the ratio R_{iso} of all molecules containing excess ^{13}C to the unlabeled molecules. The data shown here for R_{iso} do not directly reflect the ratio of BVOC recently synthesized/not recently synthesized by the plant. R_{iso} gives only a lower limit for this quantity because R_{iso} depends on the duration of foregoing $^{13}CO_2$ exposure. There may be pools of the BVOC itself or of its precursors and it therefore takes time before the degree of labeling has reached its maximum. As we had to restrict the duration of $^{13}CO_2$ exposure, the degree of labeling may have been below the maximum.

As well as the CO_2 taken up by the plant, there may be other carbon sources for BVOC synthesis[33]. Even after reaching a steady state for the degree of labeling, R_{iso} reflects only the fraction of BVOC recently synthesized from CO_2.

2.4.2 Labeling of aromatic BVOC

The emissions of BVOC synthesized in the phenylpropanoid pathway[34-36] are well known. As biosynthetic activity of the phenylpropanoid pathway is induced by stress[32, 34] such emissions are not permanent but appear periodically. Besides BVOC synthesized within the phenylpropanoid pathway, plants may also emit other aromatic BVOCs. In several cases the biosynthetic pathways of their synthesis are unknown. One example is toluene[37].

Toluene is emitted in large amounts from anthropogenic sources and impurities in ambient air used in plant experiments may be misleading and incorrectly suggest biogenic toluene emissions. As for all other VOC emitted from anthropogenic sources, toluene contains carbon with a natural ^{13}C abundance (~1% ^{13}C of C atoms). Hence, labeling of toluene observed during or after exposing plants to $^{13}CO_2$ is indicative of a biogenic origin[37]. This also applies to xylene and phenol. We term excess ^{13}C in the emitted compound during or after $^{13}CO_2$ exposure of plants as labeling. For comparison we also list the labeling of methyl salicylate (MeSA) which is a well-known aromatic BVOC produced in the phenyl-propanoid pathway (Supplementary Table 3).

Supplementary Table 3: Overview of R_{iso} as measured for toluene, xylene, phenol, and MeSA emitted from different species after 3 – 8 hours of $^{13}CO_2$ exposure. - = insignificant labeling due to small signal or overlap with other BVOC. n.o. = not observed. Natural ^{13}C content of ~ 1% per carbon atom was always subtracted.

Species	Toluene	Xylene	Phenol	MeSA
Scots pine / pathogen	0.015	0.32	n.o.	0.03
Scots pine / insect	1.45	6.22	-	0.54
Ponderosa pine	-	1.39	n.o.	0.94
Silver birch	5.44	n.o.	n.o.	2.0
Poplar / O_3	2.0	1.9	1.7	4.3
English oak / mildew	n.o.	n.o.	-	2.4
Tomato / O_3	0.2	n.o.	-	1.2

The degree of labeling varied from plant to plant. In some cases no labeling was measurable; in other cases more than half of the emitted aromatic BVOC contained excess ^{13}C (R_{iso}= 1 means that 50% of the molecules contain excess ^{13}C). Even for individuals of the same species exposed to different stresses, differences in the degree of labeling were found.

Although labeling is strongly indicative of the biotic origin of an emitted compound, care has to be taken because artefacts on adsorption tubes might cause misinterpretation of the results[38]. To exclude this possibility, measurement with Proton-Transfer Mass Spectrometer (PTR-MS, Ionicon) was conducted using a poplar individual with strong emissions of toluene, xylene, and phenol. Because PTR-MS is used without pre-concentration devices, no such

artefacts can appear. All three labeled compounds were detected by PTR-MS indicating that the respective compounds were not produced by artefacts on adsorption tubes. We therefore believe that the labeling of toluene, xylene, and phenol is due to the incorporation of ^{13}C during biosynthesis and not due to artefacts. Supplementary Figure 6 shows parts of the mass spectra of xylene and phenol as well as those of MeSA as measured ~ 3.5 h after starting a $^{13}CO_2$ exposure of poplar.

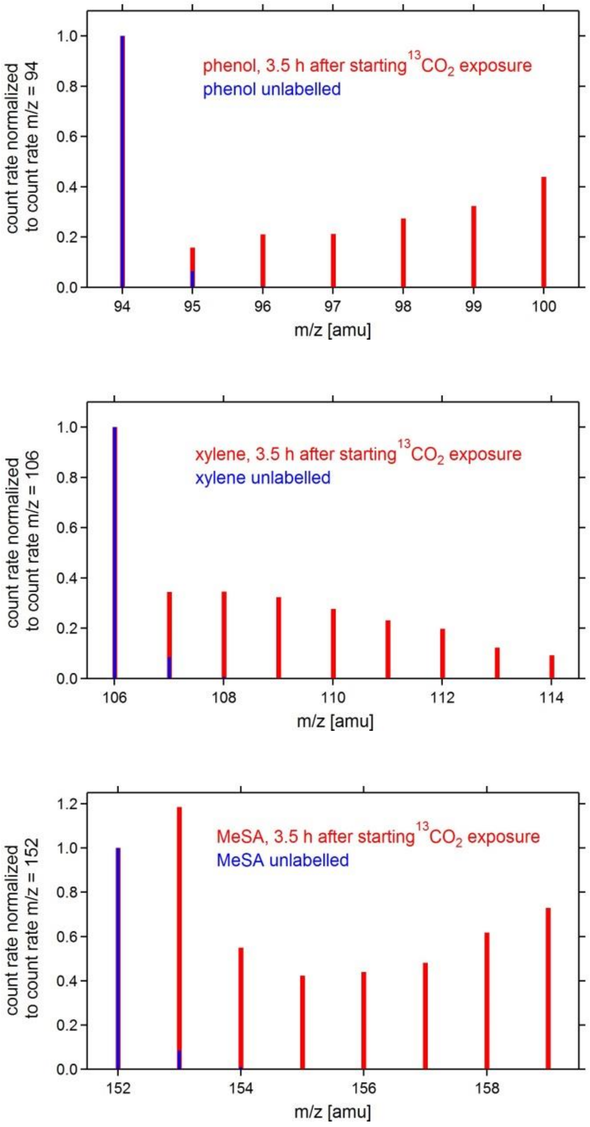

Supplementary Figure 6: Labeling of phenol, xylene, and MeSA 3.5 hours after starting $^{13}CO_2$ fumigation of poplar. Plotted are count rates of mole peaks m/z to (m+n)/z with n = number of

carbon atoms in the molecule normalized to the count rates of the mole peaks. Phenol: mole peak at m/z = 94, n= 6, xylene: mole peak at m/z =106, n =8, MeSA: mole peak at m/z = 152, n= 8,). For comparison the mass spectra obtained for the respective BVOC before exposing the plants to $^{13}CO_2$ are shown (blue bars). The differences in degree of labeling as well as in the distribution of ^{13}C are obvious.

From Supplementary Figure 6 it is obvious that phenol, xylene, and MeSA all still contained molecules consisting exclusively of ^{12}C even after fumigation with $^{13}CO_2$ for 3.5 hours. Thus, all these compounds may either have unlabeled storage pools, or they can be synthesized using either newly fixed (labeled) or stored (unlabeled) precursors. MeSa for example may be derived in part from unlabeled pools of phenylalanine. Apart from that the degree of labeling, the distribution of ^{13}C atoms in the emitted BVOC differed between the three compounds.

As mentioned by Heiden et al.[37] the labeling of toluene emitted from sunflower and Scots pine differs from that of MeSA. Heiden et al. concluded from this that toluene is not synthesized within the phenyl-propanoid pathway. Similarly, the labeling of xylene is different from that of MeSA. We therefore conclude that xylene is also not produced in this biosynthetic pathway. However, for phenol we found a similar $^{13}C/^{12}C$ pattern as for MeSA. In contrast to toluene and xylene, phenol may be produced in the phenyl-propanoid pathway.

The investigated plants emitted also other aromatic compounds than toluene, xylene, phenol, and MeSA. In several cases such aromatic compounds were labeled when the plants were exposed to $^{13}CO_2$, as shown in Supplementary Table 4.

Supplementary Table 4: List of aromatic compounds observed in the emission pattern of plants exposed to $^{13}CO_2$. Plants: SP/P = Scots pine pathogen attack, SP/I = Scots pine insect attack, PP = Ponderosa Pine without purposeful stress application, SB = Silver birch without purposeful stress application, PL/O_3 = Poplar after ozone exposure, EO/M = English oak, powdery mildew infestation TM/O_3 = Tomato after ozone exposure.

+ = significant labelling. - = either insignificant low labelling or overlap with other peaks. n.o.

BVOC	Elemental formula	SPP	SPI	PP	SB	PL/O3	EO/M	TM/O3
Benzyl alcohol	C_7H_8O	+	n.o.	-	n.o.	n.o.	n.o.	+
Cresol*	C_7H_8O	+	n.o.	-	n.o.	n.o.	n.o.	n.o.
Salicyl aldehyde	$C_7H_6O_2$	+	-	-	n.o.	n.o.	n.o.	n.o.
Benzenacetaldehyde	C_8H_8O	n.o	n.o.	n.o.	n.o.	n.o.	+	-
Methyl benzoate	$C_8H_8O_2$	+	-	n.o.	n.o.	+	n.o.	n.o.
Phenylethyl alcohol	$C_8H_{10}O$	n.o	n.o.	n.o.	n.o.	n.o.	+	+
Chavicol	$C_9H_{10}O$	n.o	n.o.	n.o.	n.o.	n.o.	n.o.	+
Benzylacetate	$C_9H_{10}O_2$	+	+	n.o.	n.o.	n.o.	n.o.	n.o.
Ethylbenzoate	$C_9H_{10}O_2$	+	-	n.o.	-	n.o.	-	n.o.
Methyl cinnamate	$C_{10}H_{10}O$	+	n.o.	+	n.o.	n.o.	n.o.	n.o.
Anethol*	$C_{10}H_{12}O$	+	-	-	n.o.	n.o.	n.o.	n.o.
Cuminal	$C_{10}H_{12}O$	+	n.o.	-	+	n.o.	n.o.	n.o.

= not observed in the emissions of the respective plant.

* = different isomers

Our labeling experiments confirm that many different aromatic BVOC may be emitted from plants. As expected for stress induced emissions, emission strengths as well as emission patterns vary from plant to plant and consequently, not all plants exhibit significant emissions. Considering the diversity of aromatic BVOCs it is not surprising that toluene, xylene or phenol can also be emitted by plants. Considering furthermore the multiplicity of biotic and abiotic

stressors for plants it is quite possible that vegetation is indeed a significant source of aromatic BVOC.

In most cases the emission rates of aromatic BVOC observed in the labeling experiments were lower than those of monoterpenes or isoprene, but in some cases significant emissions were found. In particularly, for poplar, emissions of aromatic BVOC were often high when the plants suffered from moderate stress and were even higher than those of isoprene (see Kiendler-Scharr et al.[39]).

Supplementary

References

1. Crocoll, C., Asbach, J., Novak, J., Gershenzon, J. & Degenhardt, J. Terpene synthases of oregano (Origanum vulgare L.) and their roles in the pathway and regulation of terpene biosynthesis. *Plant Mol. Biol.* **73**, 587-603 (2010).

2. Kant, M. R., Bleeker, P. M., Wijk, M. V., Schuurink, R. C. & Haring, M. A. Plant volatiles in defence. *Adv. Bot. Res.* **51**, 613-666 (2009).

3. Schiestl, F. & Cozzolino, S. Evolution of sexual mimicry in the orchid subtribe orchidinae: the role of preadaptations in the attraction of male bees as pollinators. *BMC Evolutionary Biology* **8**, 27 (2008).

4. Jardine, K. *et al*. Volatile organic compound emissions from Larrea tridentata (creosotebush). *Atmos.Chem.Phys* **10**, 12191-12206 (2010).

5. Cappellin, L. *et al*. On data analysis in PTR-TOF-MS: From raw spectra to data mining. *Sensors Actuators B: Chem.* **155**, 183-190 (2011).

6. Fall, R., Karl, T., Hansel, A., Jordan, A. & Lindinger, W. Volatile organic compounds emitted after leaf wounding: On-line analysis by proton-transfer-reaction mass spectrometry. *Journal of Geophysical Research: Atmospheres (1984–2012)* **104**, 15963-15974 (1999).

7. Harley, P., Guenther, A. & Zimmerman, P. Effects of light, temperature and canopy position on net photosynthesis and isoprene emission from sweetgum (Liquidambar styraciflua) leaves. *Tree Physiol.* **16**, 25-32 (1996).

8. Kleist, E. *et al*. Irreversible impacts of heat on the emissions of monoterpenes, sesquiterpenes, phenolic BVOC and green leaf volatiles from several tree species. *Biogeosciences* **9**, 5111-5123 (2012).

9. Li, T., Holopainen, J. K., Kokko, H., Tervahauta, A. I. & Blande, J. D. Herbivore-induced aspen volatiles temporally regulate two different indirect defences in neighbouring plants. *Funct. Ecol.* **26**, 1176-1185 (2012).

10. Jardine, K., Wegener, F., Abrell, L., van Haren, J. & Werner, C. Phytogenic biosynthesis and emission of methyl acetate. *Plant, Cell Environ.* **37**, 414-424 (2014).

11. Worton, D. *et al*. Origins and composition of fine atmospheric carbonaceous aerosol in the Sierra Nevada Mountains, California. *Atmos.Chem.Phys* **11**, 10219-10241 (2011).

12. Kaser, L. *et al*. Comparison of different real time VOC measurement techniques in a ponderosa pine forest. *Atmos.Chem.Phys* **13**, 2893-2906 (2013).

13. Misztal, P. *et al*. Direct ecosystem fluxes of volatile organic compounds from oil palms in South-East Asia. *Atmospheric Chemistry & Physics* **11**, 8995-9017 (2011).

14. Park, J. H. *et al*. Active atmosphere-ecosystem exchange of the vast majority of detected volatile organic compounds. *Science* **341**, 643-647 (2013).

15. Mak, J., Su, L., Guenther, A. & Karl, T. A novel Whole Air Sample Profiler (WASP) for the quantification of volatile organic compounds in the boundary layer. *Atmospheric Measurement Techniques* **6**, 2703-2712 (2013).

16. Yassaa, N. *et al*. Evidence for marine production of monoterpenes. *Environmental Chemistry* **5**, 391-401 (2008).

17. Yassaa, N., Colomb, A., Lochte, K., Peeken, I. & Williams, J. Development and application of a headspace solid-phase microextraction and gas chromatography/mass spectrometry method for the determination of dimethylsulfide emitted by eight marine phytoplankton species. *Limnology and Oceanography: Methods* **4**, 374-381 (2006).

18. Graus, M., Müller, M. & Hansel, A. High Resolution PTR-TOF: Quantification and formula confirmation of VOC in real time. *J. Am. Soc. Mass Spectrom.* **21**, 1037-1044 (2010).

19. Karl, T. *et al*. Emissions of volatile organic compounds inferred from airborne flux measurements over a megacity. *Atmos.Chem.Phys* **9**, 271–285 (2009).

20. Adams, R. P. in *Identification of essential oil components by gas chromatography/mass spectroscopy.* 804 (Allured Publishing Corporation, Carol Stream, IL, 2007).

21. Heiden, A., Kobel, K., Langebartels, C., Schuh-Thomas, G. & Wildt, J. Emissions of oxygenated volatile organic compounds from plants Part I: emissions from lipoxygenase activity. *J. Atmos. Chem.* **45**, 143-172 (2003).

22. Mentel, T. F. *et al*. Photochemical production of aerosols from real plant emissions. *Atmos.Chem.Phys* **9**, 4387-4406 (2009).

23. Blande, J. D., Korjus, M. & Holopainen, J. K. Foliar methyl salicylate emissions indicate prolonged aphid infestation on silver birch and black alder. *Tree Physiol.* **30**, 404-416 (2010).

24. Bouvier-Brown, N. C. *et al*. Methyl chavicol: characterization of its biogenic emission rate, abundance, and oxidation products in the atmosphere. *Atmos. Chem. Phys.* **9**, 2061-2074 (2009).

25. Müller, M. *et al*. First eddy covariance flux measurements by PTR-TOF. *Atmos. Meas. Tech.* **3**, 387-395 (2010).

26. Tani, A., Hayward, S., Hansel, A. & Hewitt, C. N. Effect of water vapour pressure on monoterpene measurements using proton transfer reaction-mass spectrometry (PTR-MS). *International Journal of Mass Spectrometry* **239**, 161-169 (2004).

27. Misztal, P. K. *et al*. Large estragole fluxes from oil palms in Borneo. *Atmos. Chem. Phys.* **10**, 4343-4358 (2010).

28. Gentner, D. R. *et al*. Elucidating secondary organic aerosol from diesel and gasoline vehicles through detailed characterization of organic carbon emissions. *Proceedings of the National Academy of Sciences* (2012).

29. Sinha, V. *et al*. Air-sea fluxes of methanol, acetone, acetaldehyde, isoprene and DMS from a Norwegian fjord following a phytoplankton bloom in a mesocosm experiment. *Atmospheric Chemistry and Physics* **7**, 739-755 (2007).

30. Graus, M. *et al*. Biosphere-atmosphere exchange of volatile organic compounds over C4 biofuel crops. *Atmos. Environ.* (2011).

31. Fares, S. *et al*. Biogenic emissions from Citrus species in California. *Atmos. Environ.* (2011).

32. Kleist, E. *et al*. Irreversible impacts of heat on the emissions of monoterpenes, sesquiterpenes, phenolic BVOC and green leaf volatiles from several tree species. *Biogeosciences* **9** (2012).

33. Schnitzler, J. P. *et al*. Contribution of different carbon sources to isoprene biosynthesis in poplar leaves. *Plant Physiol.* **135**, 152-160 (2004).

34. Colquhoun, T. A. *et al*. A petunia chorismate mutase specialized for the production of floral volatiles. *The Plant Journal* **61**, 145-155 (2010).

35. Heiden, A. *et al*. Emission of volatile organic compounds from ozone-exposed plants. *Ecol. Appl.* **9**, 1160-1167 (1999).

36. Shulaev, V., Silverman, P. & Raskin, I. Airborne signalling by methyl salicylate in plant pathogen resistance. *Nature* **385**, 718-721 (1997).

37. Heiden, A. *et al*. Toluene emissions from plants. *Geophys. Res. Lett.* **26**, 1283-1286 (1999).

38. Niinemets, Ü. *et al*. Estimations of isoprenoid emission capacity from enclosure studies: measurements, data processing, quality and standardized measurement protocols. *Biogeosciences* **8** (2011).

39. Kiendler-Scharr, A. *et al*. Isoprene in poplar emissions: effects on new particle formation and OH concentrations. *Atmospheric Chemistry & Physics* **12** (2012).
